# Temperature Sensor Denoising Algorithm Based on Curve Fitting and Compound Kalman Filtering

**DOI:** 10.3390/s20071959

**Published:** 2020-03-31

**Authors:** Yang Zhang, Rong Wang, Shouzhe Li, Shengbo Qi

**Affiliations:** 1 The College of Engineering, Ocean University of China, Shandong 266100, China; zhyang@stu.ouc.edu.cn (Y.Z.);; 2 College of Letters and Science, University of Wisconsin-Madison, Madison, WI 53711, USA

**Keywords:** least squares method, CKSF, wavelet transform, temperature sensor, noise, noise variance

## Abstract

One of the most important ocean water parameters in world ocean observations is temperature. In the application of high-precision ocean sensors, there are often various interferences and random noises. These noises will cause the linearity of the sensor to change, and it is difficult to estimate the statistical characteristics, and the results will deviate from the real temperature. Aiming at the problems in the application, this paper proposes a compound Kalman smoothing filter (CKSF) algorithm based on least square curve fitting. This algorithm first analyzes the system model of the sensor, uses the least square method to fit the theoretical data and eliminate the non-linear factors caused by system itself, then estimates the statistical characteristics of the noise required by modeling, using the wavelet transform method to track the change of noise in real time and to accurately estimate the noise variance. Finally, a compound filtering method including wavelet transform and Kalman smoothing filtering is used as the main denoising algorithm, which is more accurate than a single Kalman filtering result. The algorithm is applied to the temperature measurement process of the ocean temperature sensor. The results show that the accuracy and stability of the sensor are improved.

## 1. Introduction

The sensor is a detection device that can convert the detected values into an electrical signal. It is a key device to achieve complex control in the field of mechanical control and automation design. In particular, temperature sensors have important applications in the fields of water quality prediction and industrial control [1,2], and temperature is also an important marine physical parameter. According to relevant studies [3], a deviation of 0.25 °C in seawater temperature will cause changes in water oxygen content, threatening the survival of some marine organisms. If the temperature fluctuation is large, it will cause natural meteorological disasters, and most physical parameters and detection instruments require temperature compensation. Therefore, high precision and stable temperature sensors are very important in research [4]. However, the complex environmental noise and system errors often limit the improvement of sensor accuracy. In the application, various filtering algorithms are often used to improve stability and precision, such as low-pass filtering, which can reduce the ripple caused by stable environmental noise, but a single hardware filtering method cannot adapt to the complex and changeable marine environment and time-varying noise. Therefore, in many sensors (e.g., the surface acoustic wave sensors, temperature sensor etc.) design processes often need to add intelligent algorithms to achieve sensor denoising.

At present, the more practical algorithms for temperature sensor denoising are the methods proposed in [2,5]. Although these algorithms can improve the accuracy of temperature sensors, the marine environment is often changeable and complex, so a single algorithm is difficult to adapt. In addition, the Kalman filter model needs to estimate the statistical characteristics and system characteristics of the noise, especially the estimation of the covariance of the noise, which is very important for the output accuracy of the model. In [6,7], different algorithms were proposed, and most of them used complex mathematical methods to solve the statistical characteristics of noise. The application background is complex, and it is suitable for multivariate signal processing.

In this paper, a compound Kalman filtering method is proposed, which has a higher robustness and accuracy than the single Kalman filter method. It mainly consists of two parts: the least square method for fitting and wavelet transform for noises variance estimation. This focuses on the problem of estimating the statistical characteristics of noise during non-stationary temperature measurement, using the wavelet transform method. This method has the advantage of local signal analysis and optimization, and predicts noise changes in real time [8]. We then focus on the non-linear characteristics of the sensor caused by noise, using the least square method for curve fitting to get mathematical relationship between resistance and temperature, selecting the appropriate degree of fit to eliminate fault values. The output of the system after curve fitting could be only disturbed by the environmental noise, reducing the influence of system-inherent errors, and further improving the stability and accuracy of the compound Kalman smoothing filter (CKSF) model. Using smoothing filters after Kalman filtering can make the results of Kalman filtering more accurate.

The main difference between the method in this paper and the traditional Kalman denoising algorithm is that the observed noise variance *P(k)* in Equation (10) is not determined in advance based on empirical values, but is estimated based on the signal value of the measured noise. This proves that the variance of the observation noise used in the calculation is basically the same as the variance of the observation noise of the actual system. Even if the noise of the actual system changes due to some interference, the variance estimated by the algorithm can accurately follow the variation of the noise of the observed signal. In order to verify the algorithm, the article takes a temperature sensor based on platinum (Pt) thermal resistance. However, during the actual measurement in the seawater for seven days, we found that the sensor is affected by environmental noise and system noise, which caused the measured temperature value to fluctuate greatly and inaccurately reflect the true temperature. The comparative experimental results show that the optimized design greatly improves the measurement accuracy.

## 2. CKSF Denoising Algorithm Analysis

### 2.1. Kalman Filter Model of Temperature Sensor

**Figure 1:**
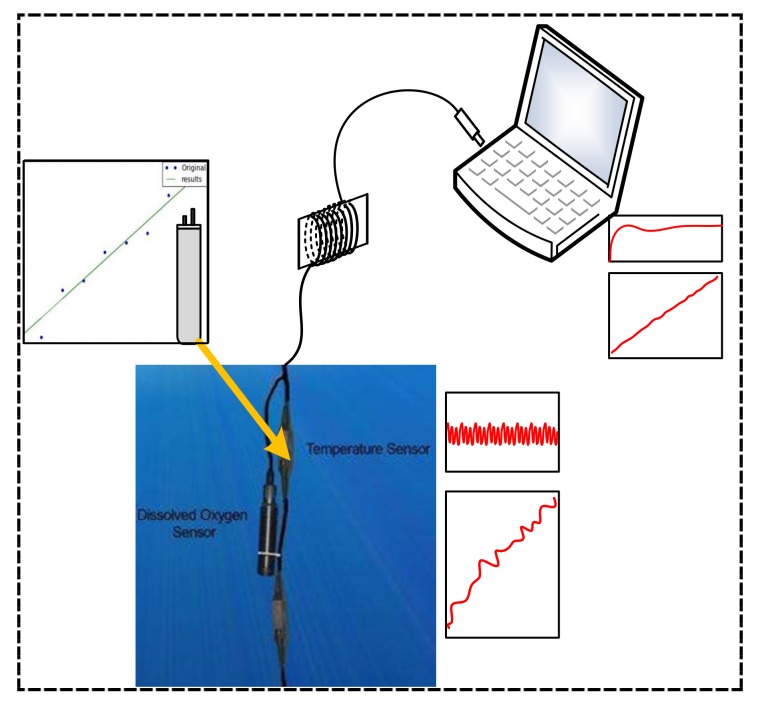
The algorithm application diagram, the upper left picture is the temperature sensor data fit, and the red waveform is a schematic diagram of the collected temperature data.

The Kalman filter includes two parts: the prior estimation and the posterior estimation of the state [9]. Based on the sensor system, this article designs a Kalman smoothing filter based on Kalman estimation, which mainly consists of noise variance characteristics estimation and Kalman filtering and data averaging. By comparing the single Kalman filter model and the CKSF algorithms, we show that the algorithm in this paper can effectively reduce the 0.006 °C (an average of errors) error. The actual application of the sensor system is shown in Figure 1.

The main advantage of the platinum thermal resistance temperature sensor (PTS) over thermocouples and thermistors is that PTSs have good linearity, so the sensor discrete control system can be approximated as:

(1) $$\left\{\begin{array}{c} x\left(k\right)=F\left(k-1\right)x\left(k-1\right)+W\left(k-1\right)\\ y\left(k\right)=B\left(k\right)x\left(k\right)+V\left(k\right) \end{array}\right.$$

In Equation (1), *F(k-1), B(k)* is the state transition matrix of the sensor system. If the sensor is a temperature sensor, $X(k)\in R^{n}{}^{\times 1}$ is defined as the temperature value at the moment k; *y(k)* is the observation value; *W(k-1)* and *V(k)* are the state noise and observation noise of the temperature sensor, the statistical characteristics of this noise are random and uncertain in applications. The method of wavelet transform is used to estimate the variance characteristics of this kind of uncertainty noise. It is assumed here that the noise is White Gaussian Noise, and the statistical characteristics of the noise satisfy:

(2) $$\left\{\begin{array}{c} E\left[W\left(k-1\right)\right]=E\left[V\left(k\right)\right]=0\\ Cov\left[W\left(k-1\right)\right]=Q\left(k-1\right)\\ Cov\left[V\left(k\right)\right]=R\left(k\right) \end{array}\right.$$

where *Q(k-1)* and *R(k)* represent the variance of process noise *W(k-1*) and measurement noise *V(k)*. Therefore, the sampling temperature value (T) output by the system at continuous moments is:

(3) $$Y\left(k\right)=\overset{k}{\underset{n=1}{\sum }}y\left(n\right)=\left(T1+T2+\ldots +Tn\right)$$

The specific algorithm steps of using the improved Kalman filtering algorithm to the temperature sensor are:

The estimated value of the temperature at time *k-1* is $\hat{x}\left(k-1\right)$, then the further estimation (prior estimate) of the state at moment *k* is the Equation (4) and the estimated covariance is Equation (5), $\hat{x}\left(k|k-1\right)$ indicates that the temperature at time k is estimated using time k-1.

(4) $$\hat{x}\left(k|k-1\right)=F\left(k-1\right)\hat{x}\left(k-1\right)$$

(5) $$P\left(k|k-1\right)=E\left\{\left[x\left(k-1\right)-\hat{x}\left(k-1\right)\right]{\left[x\left(k-1\right)-\hat{x}\left(k-1\right)\right]}^{T}\right\}$$

**Figure 2:**
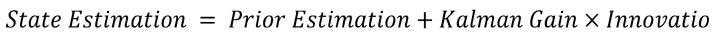
Schematic of using state recursive estimation to correct errors.

Equations (4) and (5) complete the prior rough estimation of the state from *k-1* to *k*. Obviously, different noises may be added in this process [10], and the predicted value may not completely reflect the true temperature. Using the state recursive estimation, the estimated value after error correction is shown in Figure 2. where the innovation is usually represented by *a(k)*, indicating the difference between the predicted value and the actual value, and Kalman gain *G(k)* indicates that this part of the innovation information occupies proportions [11,12]. So, based on the estimation method in Figure 2, we can deduce:

(6) $$\left\{\begin{array}{c} \hat{x}\left(k\right)=\hat{x}\left(k-1\right)+G\left(k\right)a\left(k\right)\\ a\left(k\right)=y\left(k\right)-H\left(k\right)\hat{x}(k|k-1) \end{array}\right.$$

where *y(k)* indicates the state observation measurement of the posterior estimation, and *H(k)* indicates the state transition at time k. In addition, the state value *x(k)* and observed value *y(k)* are both the environment temperature value (°C). There is no state transition. Assume *H(k) = I* for ease of calculation.

Moreover, the covariance of the innovation in the temperature estimation process can be obtained:

(7) $$P\left(k\right)=E\left[a\left(k\right)a{\left(k\right)}^{T}\right]=E\left[x\left(k\right)+V\left(k\right)-\hat{x}\left(k|k-1\right)\hat{x}{\left(k|k-1\right)}^{T}\right]=R\left(k\right)+P_{e}\left(k\right)$$

Because of the state prediction and observation noise correlation coefficients ρ satisfies $\rho \left[V\left(k\right),e\left(k\right)\right]=0.$ The innovation variance can be written as the result of Equation (7), $E[\hat{e}(k)\hat{e}{\left(k\right)}^{T}]=P_{e}(k)$ is the state prediction error covariance. Establishing a posterior estimation model, the estimated state results need be evaluated, so the error in the state estimation process is defined as

(8) $$\left\{\begin{array}{ll} e(k) & =x(k)-\hat{x}(k)\\ & =[I-G(k)H(k)]e(k|k-1)\\ P(k) & =[I-G(k)H(k)]P(k|k-1) \end{array}\right.$$

where *e(k|k-1)* and *P(k)* represents the state prediction error and the covariance of the state estimation error for the posterior estimation model, *e(k)* represents the deviation between real value and predictive value at the moment of k.

**Figure 3:**
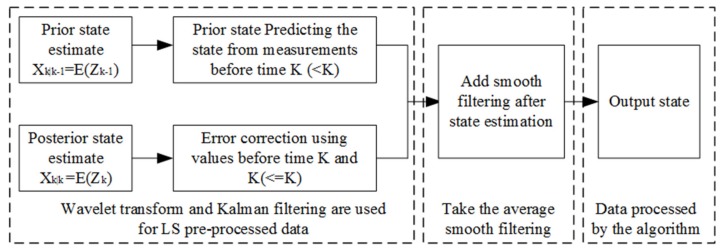
The measurement principle block diagram of the improved Kalman filter algorithm, including two parts of design data processing by algorithm and average value filtering (The method of averaging the observations is called smooth filtering in diagram.).

Equations (2) to (8) are the modeling processes of improved Kalman filter estimation. Because the Kalman filter algorithm is a recursive algorithm based on prior and posterior estimates [9], the Kalman filter algorithm can only be used for state estimation at moment *k* and *k-n(n<k)*. The improved algorithm adds an average value of smooth filtering and the wavelet transform method, which reduces the data error and improves the accuracy of the state estimation, as shown in Figure 3.

However, in practical problems, CKSF modeling requires two important parameters, namely the Kalman gain and the initial temperature value of the sensor observation. In this paper, using the Kalman recursive least squares estimation to solve the Kalman gain, *Z(k)* is defined as the objective function, as shown in Equation (9):

(9) $$\begin{array}{ll} Z(k) & =E[e(k)e{\left(k\right)}^{T}]\\ & =E[tr(e(k)e{\left(k\right)}^{T})] \end{array}$$

where tr[*e(k)e(k)^T^*] represents the trace of matrix. The objective function *Z(k)* is minimized using the Lagrange multiplication and matrix inversion method [13] to deduce the proportion of innovation information, that is, Kalman gain *G(k)*. Here, assume H to be the identity matrix.

(10) $$G(k)=P(k\left|k-1\right.)H^{T}{\left[HP(k\left|k-1\right.)H^{T}+P(k)\right]}^{-1}$$

The second parameter is to solve the initial temperature values. The CKSF algorithm needs to assume the initial state of the system and the initial estimation error covariance. Generally, the initial estimation error covariance of the system is not set to zero because it will cause the Kalman filtering algorithm to trust the initial prediction state too much. As a result, the estimation error of the algorithm will be too large. Based on the empirical model [10], the initial estimation error covariance of the temperature sensor is:

(11) $$P(0)=E\{[x(0)-\hat{x}(0)]{\left[x(0)-\hat{x}(0)\right]}^{T}\}$$

### 2.2. Estimation of Noise Variance During Temperature Observation

The modeling assumes that the noise in the temperature sensor observation process is Gaussian white noise, but the statistical characteristics and distribution of the noise in the actual process are unknown. If the statistical characteristics of the noise change significantly, the mean square error of the improved Kalman filter solution has deviated from the real situation (real temperature values) and cannot reflect the accuracy of the state estimation, although the effect on the result may be small for a period of time [12]. That is to say the ideal model observation data is a mixture of signal and noise [14] and not suitable for practical applications.

When noise and signal are mixed, the analysis process becomes complicated. At present, there are many methods for separating noise and signal, such as the commonly used oblique projection filtering, particle filter, and blind separation algorithm [15,16]. However, in the Kalman smoothing filter, only the statistical characteristics of the noise needs to be known, and the noise and signals need not be accurately separated, therefore using the wavelet transform method to estimate the statistical characteristics of the noise. The wavelet transform can focus on the signal detailed aspect and realize the precise local optimization. Using this method, we can separate the high-frequency noise that mainly affects the actual measurement data, and solve the variance of the high-frequency noise.

The detection temperature values of the sensor with random noise is approximated by a polynomial expansion method with an error *f(k)*:

(12) $$y\left(k\right)=b_{0}k_{0}+b_{1}k_{1}+\cdots +f\left(k\right)$$

Yielding the Equation (13) by the linear transformation of the wavelet function:

(13) $$\psi_{a,\tau }(k)=a^{-0.5}\psi (\frac{k-\tau }{a})$$

Equation (13) describes the relationship between the zoom (a) and translation (*τ*) transformed from the wavelet mother function and the mother function transformation sequence $\psi_{a,\tau }(k)$.

Using wavelet transform to process the temperature sensor observation data *y(k)*:

(14) $$W_{tl}\left(a,\tau \right)=y\left(k\right)\times \psi_{a,\tau }\left(k\right)=W_{t}\left(a,\tau \right)+W_{l}\left(a,\tau \right)$$

The $W_{tl}\left(a,\tau \right)$ represents the function after wavelet transform. The $W_{t}\left(a,\tau \right)\;\mathrm{and}\;W_{l}\left(a,\tau \right)$ denotes the approximate part and the detail part of the wavelet coefficients. Choosing the suitable vanishing moments of the mother function, the wavelet transform will enhance the ability of the observed signal to suppress the source signal, so that only the noise component $W_{l}(a,\tau )$ is retained [17].

**Figure 4:**
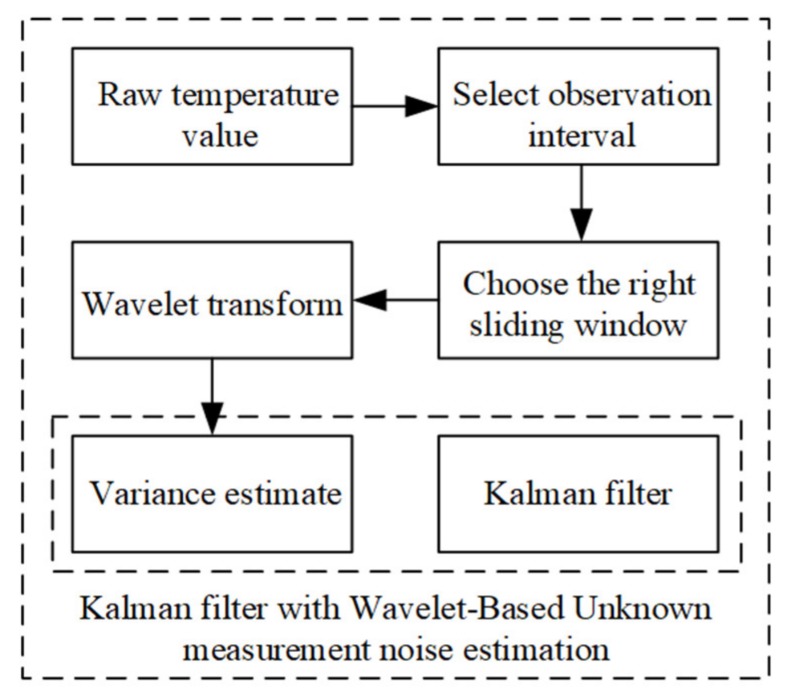
The flow diagram of the wavelet transform algorithm to solve the noise variance.

If the state value of the temperature is the piecewise polynomial within a period of time, a suitable observation interval is selected so that the vanishing moment of the wavelet mother function is greater than the order of the highest polynomial in the piecewise polynomial. The implicit noise variance in the process of measuring the temperature value can be presented by using the median estimation of the approximate part of the wavelet coefficients in the wavelet transform [18], which is Equation (15):

(15) $$D_{\delta }(k)\approx \frac{MAD}{0.6745}$$

where the MAD is the median value of the high-frequency sub-band wavelet coefficient amplitudes contained in all decomposition layers after wavelet transform [19]. The *D_δ_ (k)* is the noise global variance. In summary, the steps of estimating the variance of noise by wavelet transform are shown in Figure 4.

### 2.3. Sensor Resistance and Temperature (R_T) Fitting Model

The thermal resistance temperature sensor is mainly used in this paper. The linearity of platinum thermal resistance is better than that of a thermistor, and the measurement value between low and medium temperature measurement is stable. If the sensor is affected by noise, the linearity deteriorates and the non-linear characteristics increases. This will cause the Kalman filtering algorithm result and accuracy to deviate from the ideal values. At present, the basic processing method is system linearization, such as the use of extended Kalman filter (EKF), volume Kalman filter (VKF) and other methods, which can solve nonlinear problems [20]. However, the algorithm is complicated, and the robustness of the system will be reduced [21]. For the non-linear part of the system, it only appears in the stage of interference, thus the direct linearization method is not very practical. The main method used in this paper is to fit the temperature characteristic curve by using the least square method, and then to perform the Kalman smoothing method. The data results show that the system is stable and the temperature observation value is closer to the real temperature.

Using the least square method fitting for the curve of R_T, the discrete state equation of the observation is the Equation (1). For a given state value *x(k)*, there is a corresponding $\hat{y}\left(k\right)$ in the state estimation, the estimated value is often different from the observed value *y(k)*. There is a deviation between them. The sum of the squared deviations is defined as:

(16) $$J(k)=\sum_{i=1}^{n}[y(k)-\hat{y}(k)]{\left[y(k)-\hat{y}(k)\right]}^{T}$$

In the state estimation, the state estimation of the regression equation should be as accurate as possible. According to the minimum mean square error (MMSE) methods, the partial derivatives for *J(k)* need satisfy [22]:

(17) $$\frac{\partial }{\partial x_{i}}J(k)=0$$

**Figure 5:**
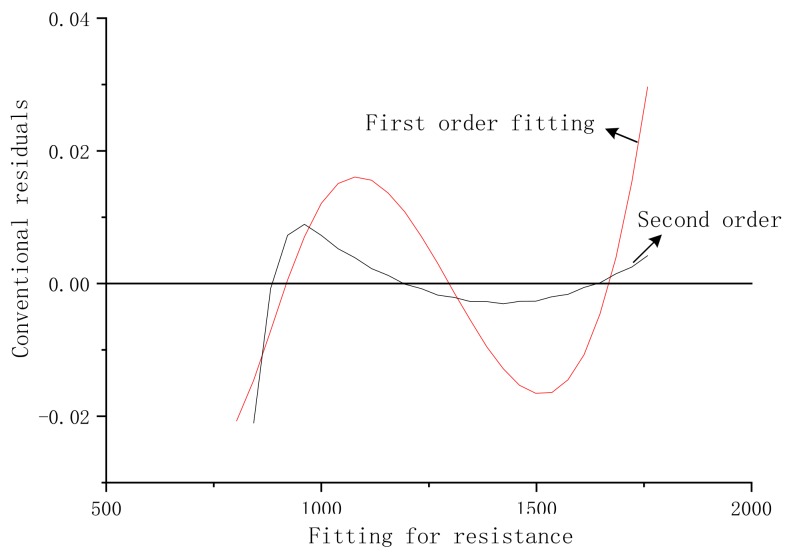
Comparison chart of the effect of first-order and second-order fitting using the least square method.

In this paper, we perform a first- and second-order fitting on the temperature data. By comparing the conventional residuals, as shown in Figure 5, the second-order fitting has the best effect.

## 3. Experimental Data Analysis and Discussion

### 3.1. Temperature Sensor Selection and Application 

The data were measured by using a temperature sensor, and a series of actual interference and theoretical noise were added. The validity of the CKSF algorithm is verified by comparing the processed data with the original data. In this experiment, the host computer software was written by using C SHARPE and Visual Studio 2019 software (Microsoft Corporation) to obtain the resistance and temperature values measured by the temperature sensor.

The temperature sensor used in the experiment is a platinum thermal resistance (PTR) type, degrees B (technical parameter), the values of temperature coefficient of resistance (TCR) is 0.003851 and the temperature calculation follows the empirical Equation (18):

(18) $$R(t)=R_{0}(1+At+Bt^{2})$$

where A and B are constants, *R_0_* is the resistance values at 0 °C.

### 3.2. The Analysis of Least Squares Method Results 

This analysis is based on Section 2.3, using the least squares second-order fitting to calculate the value of the constant A and B in Equation (18). The resistance data of the least squares fitting is derived from the output of the precision resistance box. This calibration method can greatly improve the accuracy of the sensor compared to directly using the resistance parameters in the technical manual (In the technical manual of PTR, there are general reference values of A and B.). As the PTR sensors use different A and B parameters, Table 1 shows the comparison between using second order fitting methods and sensor manual parameters. 

**Figure 6:**
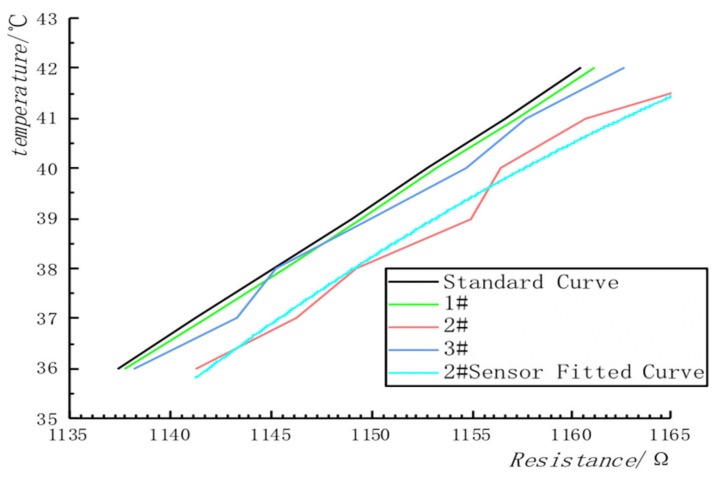
Comparison chart of the effect of first-order and second-order fitting using the least square method. The resistance on the way is the resistance of the thermistor.

In addition to the system error reflected in Table 1, in the actual measurement process, even temperature ranges with good linearity will fluctuate. These fluctuations will make the Kalman filter result inaccurate. The least square curve fitting can ensure the accuracy of the sensor’s input curve and reduce the influence of error on the observed value. In Figure 6, 1#, 2# and 3# represent the R_T data measured by three different temperature sensors. It can be seen from the experimental results that the least square method is not used to preprocess the curve, and the system error during the measurement process will cause the curve to be unstable and nonlinear.

The experimental data were preprocessed by the least square method. Figure 6 shows that the processing of the least square method (only processing the sensor 2#) can ensure that the data measured by the sensor is approximately linear, and the fitting process can make the Kalman filter model identify and predict the data more accurately.

### 3.3. The Analysis of Wavelet Transforms Results 

The data output by the sensor is obtained on the basis of the least square method to compensate the system error. However, the actual measurement process is also affected by the position of the sensor in the liquid, the environment (humidity, mechanical vibration, etc.) and the accuracy of the data calculation. It is very important for modeling to estimate the variance of these random noises.

In order to verify the performance of the CKSF algorithm in eliminating observation noise, we used the wavelet transform to process the observation data (here choosing the negative temperature coefficient sensor). The approximate part and the detail part are separated, and then the noise covariance is solved to estimate the preliminary statistical characteristics of the noise. According to the calculation in Paper [19], the sliding window was selected as 30S in the experiment.

**Figure 7:**
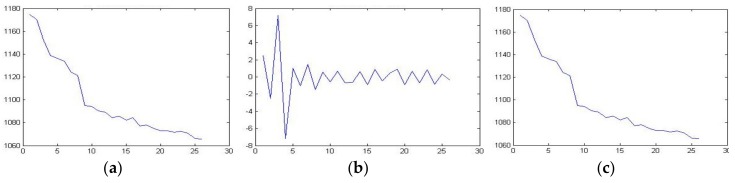
(**a**) Temperature and resistance curve with random noise; (**b**) Details of wavelet transform; (**c**) Approximate part of wavelet transform. Here, the abscissa represents the temperature value (°C), and the ordinate represents the corresponding resistance value (ohm).

**Figure 8:**
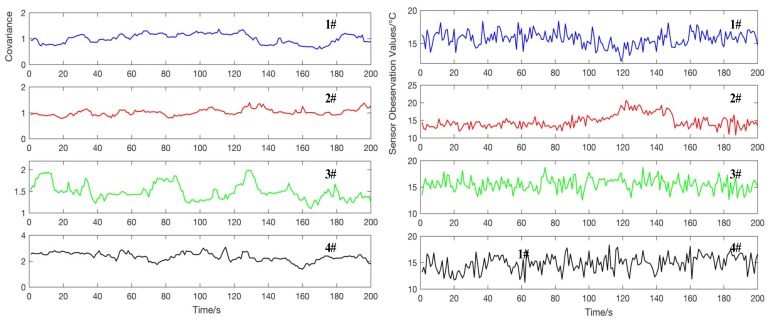
Estimation of noise variance based on wavelet transform for different sensor.

As shown in Figure 7, the wavelet transform can effectively separate the approximate part and the detailed part of the observation signal. According to the Equation (14), choosing a suitable wavelet vanishing moment for the separated noise can effectively calculate the covariance of the noise, solve the problem that the covariance of the observed noise of the Kalman filter model is difficult to estimate and have good followability for the method, identifying time-varying signals of noise change [7]. The noise covariance estimation is shown in Figure 8 (the right figure shows the monitoring values of different sensors in a constant temperature water of 15 Celsius in 200s, and the left figure shows the noise covariance estimation by using wavelet transform).

According to the analysis on the left in Figure 8, it can be observed that the wavelet transform can effectively estimate the noise covariance within a certain error range, and can track the change of noise in real time, providing a guarantee for an accurate Kalman filter model. In addition, the average filtering method is usually used to process data with large variance fluctuations.

### 3.4. Comparative Analysis with Other Algorithms 

**Figure 9:**
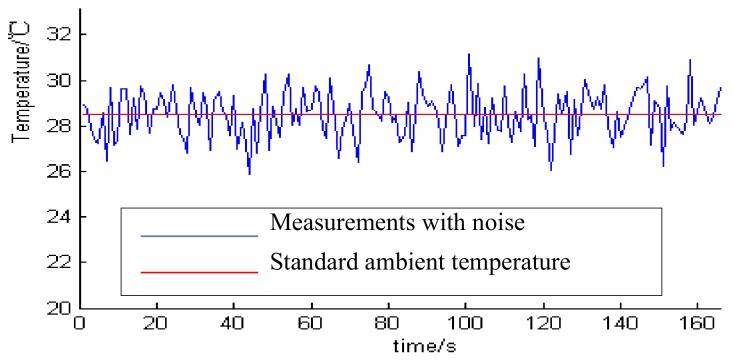
Temperature observation with random noise of constant temperature water bath.

Here, we focus on the data with random noise, using the wavelet transform method to estimate covariance. The Kalman algorithm was used to build the CKSF model. Through comparison with the unfiltered temperature value, the observation temperature is stable even with uninterrupted random noise by using CKSF algorithms. By adjusting the interval of the filtering and compensation, the high-precision output of the sensor can be effectively realized in the case of interference. The noise sources in the experiment are mainly electromagnetic vibration, humid environment, sensor position and more. The temperature data containing random noise are observed in the constant-temperature water bath (at standard atmospheric pressure, the water temperature is 28.51 degrees Celsius), as shown in Figure 9.

**Figure 10:**
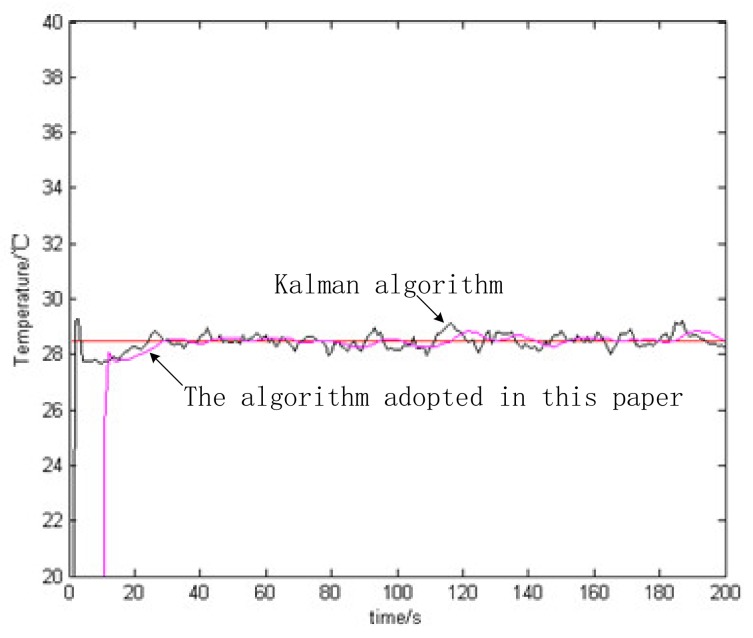
Comparison of Kalman filtering and Kalman smooth filtering.

It can be seen from Figure 9 that the artificial application of process noise will cause the measured value of the temperature sensor to deviate from the actual value, so it is inaccurate to directly use the temperature value of the disturbed sensor. Using the CKSF algorithm and the single Kalman method to process the actual observation temperature value, the algorithm in this paper is smoother than the single Kalman filter, and the experimental data is closer to the real temperature, as observed in Figure 10 and Table 2.

## 4. Conclusions

This paper mainly designs the CKSF algorithm for cases where the ocean temperature sensor is easily influenced by uncertainty and random noise, which causes the measurement data to fluctuate greatly, and deviate from the real environment temperature. 

Firstly, the noise of the sensor is analyzed, and a model using wavelet transform to estimate the statistical characteristics of the noise is proposed, including use of the least square curve fitting method to reduce the system error of the sensor. Furthermore, based on this model, we deduce the sensor data processing algorithm that compounds the Kalman smoothing filtering algorithm. Through analysis and simulation using the CKSF method, the temperature value detected by the sensor can effectively restore the actual temperature under the conditions of random and uncertain noise. Therefore, it has potential to be applied in sensor design and data processing.

## Figures and Tables

**Table 1:** The influence of temperature for different methods to obtain the parameters of A and B.

Temperature/°C ^1^	Fitting Para	Manual
20	19.6	19.3
30	29.7	29.1
40	39.9	39.0
50	49.5	49.1
60	59.9	59.4
70	69.8	69.8

^1^ Temperature represents the ambient temperature at this time; Fitting para represents using LS methods to detect temperature; Manual represents using sensor manual rate A and B value to detect temperature. For obvious comparison, we have only kept one decimal place.

**Table 2:** The data of using different algorithms to process temperature detected by ocean conductivity temperature depth sensor.

Temp1 ^1^	Error1 ^2^	Temp2 ^3^	Error2 ^4^
25.776	−2.734	28.542	0.032
28.485	−0.025	28.323	−0.187
28.416	−0.094	28.254	−0.256
28.505	−0.005	28.233	−0.277
28.495	−0.015	28.167	−0.343
28.498	−0.012	28.075	−0.435
28.483	−0.027	28.054	−0.456
28.221	−0.289	28.337	−0.173
28.36	−0.150	28.433	−0.077
28.457	−0.053	28.248	−0.262
28.199	−0.311	28.005	−0.505
28.499	−0.0011	28.016	−0.494
28.491	−0.019	28.334	−0.176
28.51	0.000	28.280	−0.23
28.445	−0.065	28.117	−0.393
28.265	−0.245	28.371	−0.139
28.322	−0.188	28.556	0.046
28.423	−0.087	28.386	−0.124
28.424	−0.086	28.365	−0.145
28.356	−0.154	28.696	0.186
28.500	−0.010	28.708	0.198
28.534	0.024	28.905	0.395
28.543	0.033	28.579	0.069
28.571	0.061	28.694	0.184
28.554	0.044	28.476	−0.034
28.566	0.056	28.610	0.100
28.57	0.060	28.685	0.175
28.589	0.079	28.664	0.154
28.597	0.087	28.629	0.119
28.562	0.052	28.429	−0.081
28.52	0.010	28.353	−0.157

^1^ Temp1 represents the sensor temperature data (°C) processed by the algorithm in this paper. ^2^ Error1 represents the error between Temp1 and the standard environment temperature. ^3^ Temp2 is the sensor data (°C) processed by the single Kalman algorithm. ^4^ Error2 is the error between Temp2 and the standard environment temperature.
